# Historical Sketch of Lithotrity

**Published:** 1829-10

**Authors:** W. B. Costello

**Affiliations:** late Assistant to the Inventor, Dr. Civiale.


### THE LONDON
### Medical and Physical Journal.

N° 368, VOL. LXII.] OCTOBER, 1829. [N° 40, *New Series.*

For many fortunate discoveries in medicine, and for the detection of numerous errors, the world is indebted to the rapid circulation of Monthly Journals; and there never existed any work, to which the Faculty, in Europe and America, were under deeper obligations than to the *Medical and Physical Journal of London*, now forming a long, but an invaluable series.—RUSH.

### ORIGINAL PAPERS, AND CASES,
### OBTAINED FROM PUBLIC INSTITUTIONS AND OTHER AUTHENTIC SOURCES.

### LITHOTRITY.

*Historical Sketch of Lithotrity.* By W. B. COSTELLO, Esq. late Assistant to the Inventor, Dr. CIVIALE.*

WHEN a great and useful discovery has been effected, and when its application begins to astonish and benefit the world, the parts which chance or industry has had in bringing it to maturity are not very easy to be precisely assigned; nor should this inquiry delay us. Lithotrity, or the method of drilling and crushing calculi in the bladder, like many other important discoveries, is an unexpected result, obtained or arrived at by a bold and acute mind, after much labour and by an indirect course.

In all inquiries which have for their object some great practical benefit, the *point de depart* is that which attracts the attention of the philosopher. How immensely has chemistry benefited by the search after the philosopher's stone! The progress of inquiry with regard to lithotrity is perfectly analogous. A pursuit equally chimerical, the dissolution of stone in the bladder, has led to this important improvement in the treatment of calculous disease; and it is indeed remarkable, amongst the numerous means employed for the cure of stone, that the most efficacious and the most safe,

* The plates referred to in various passages of this communication will be given in our next Number, with a second article from Mr. COSTELLO.—EDITORS.

namely, those by which the foreign body may be comminuted in the bladder, and which in ancient times had all but fallen within the domain of science, should have been deemed too unprofitable for direct research; whilst the idea of dissolving those concretions by means of reagents had been adopted by all, and pursued so far, till it has led us, almost without our knowledge, to the employment of mechanical agents; or, in other words, to the invention of lithotrity. Thus a means, which in the outset was merely considered as an accessory part of the treatment by reagents, has turned out to be the most simple, efficacious, and certain of all those hitherto proposed, and, by its results, entitled to rank amongst the most brilliant achievements of surgery.

The ardor with which inquiry for a better therapeutical agent in the treatment of calculous disease has been conducted, reflects high honour on those distinguished individuals who, in this and the last century, have so elaborately investigated this subject. For a moment, every thing was hoped for from modern chemistry. Those hopes, however, were never realized; the only result being a correct analysis of the concretions obtained by cutting, or after death, from the bladder or kidneys, &c. of the individuals affected. Meanwhile many eminent contemporaries were continually reducing to practice their useful meditations on the only alternative left, lithotomy, until the instruments and operative process may be considered as having attained their utmost degree of perfection.

But it is not in this century, or the last only, that the appeal of suffering humanity had been so strongly urged on the enlightened philanthropy of medical science, for the discovery of means to alleviate the protracted and grievous torments occasioned by the presence of calculus in the bladder. The liability to it suspended vaguely, and as it were *in terrorem*, over every human being, however favored by nature; and some philosophers instituted inquiries from their love of science, while others, who had felt the attacks of this internal enemy, consumed their lives in researches which, notwithstanding the strength of their desires, they were not fortunate enough to bring to a successful issue. The anxiety felt on this subject was general, and pervaded all times. A keen spirit of inquiry, traversing the most remarkable epochs in the history of the healing art, and led on by the desire of improving this very interesting branch of it, had left, it is true, thinly scattered within the boundaries of science, those rudiments which, carefully collected, combined, and rendered perfect by the genius of one man, present themselves to our view, not indeed as the developement of new and original ideas, but as a complete system, confirmatory of the speculations of philosophers, ancient and modern, establishing an operation wholly new, and which must ultimately change the face of science in regard to the treatment of calculous disease. Lithotrity, or the method of pulverising calculus in the bladder, is therefore nothing more than those obsolete and obscure hints which, in the progress of ages, human ingenuity had suggested for the cure of the most distressing ill to which "flesh is heir," brought to maturity. It is not strictly an invention, in the sense attached to the word, when the mind surprises any of nature's secrets. The want of this condition, however, by no means impairs its importance; for it still extends the limits of science, it disarms this formidable disease not only of its terrors, but of its dangers, and converts that which hitherto has been regarded as the greatest of physical ills into an occurrence which will remain terrible only by remembrance, and calling for scarcely more than an ordinary expression of sympathy.

The first attempts at extracting stone from the bladder by the natural passage were practised by the Egyptians. Prosper Albinus, who has transmitted the details of their method, informs us that he saw an Arabian physician, named Haly, deliver a Turkish commander of the stone by the following process: A pipe of wood or ivory, of the proper size and length, was introduced into the urethra; air was then gradually blown into the bladder, and, the inflation completed, a stopper was adapted tightly to the external orifice of the pipe. An attempt was then made, by the finger introduced into the anus, to force the calculus forward, and engage it in the neck of the bladder, already dilated. The stopper being then suddenly withdrawn, the air was forced out briskly, by pressing on the bladder above the pubes. When the operator had succeeded by this manœuvre in forcing the calculus into the urethra, it was brought out by suction or other appropriate means. This operation, which (as Desgenettes and Larrey assure us) is still practised in Egypt, is the most ancient of the bloodless means resorted to for the cure of stone, and though it differs widely from lithotrity, yet still it bears some analogy to it, and may be considered its *point de depart;* for, like lithotrity, it consisted in the extraction of the foreign body by the natural passage. From this practice of the Egyptians, it is evident they were well acquainted with the fact of the dilatability of the urethra; a fact still further proved by the spontaneous passage of calculi, measuring from five to six lines in diameter. Now, from the knowledge of this fact to the proposal of some means for dividing or reducing concretions exceeding this size, there is but one step.

The Greek physicians very unjustifiably neglected this method, from which much good might have been derived, and which, had it been kept more closely in view, might have contributed to the more early developement of means equally safe, but of more certain success.

Cystotomy, an operation of the highest antiquity, continued to be performed, and remained for ages the sad and sole resource of those whose acute sufferings armed them with courage against the terrors of the operation itself, and the apprehensions which the ignorance of those who performed it must have inspired. Its successes in the time of Hippocrates must have been very rare, at which period it was so fallen into disrepute as to be performed exclusively by itinerant empirics. Among these, Ammon of Alexandria had the boldness upon several occasions, where the stone was too large to be extracted by the incision made in the perineum, to employ a statuary's chisel for the purpose of breaking it into small fragments; and hence the name *lithotomos*, by which those who cut for stone were designated after him. Hippocrates supposed erroneously (and most probably his opinion was formed upon the unfavorable results obtained by those practitioners,) that all wounds of the urinary bladder were mortal. It is, therefore, matter of little wonder that his prejudices against lithotomy should have led him so far as to proscribe its performance altogether, characterizing it as a cruel and murderous operation. Nay more, he required an oath from his disciples that they should never perform an operation which he regarded as inevitably fatal. For a long time after Hippocrates, cystotomy remained degraded in the hands of the latro chirurgeons, and was utterly neglected by the schools, until the Greeks, Enos, Meges, Evelpistus, Ammon, &c. restored it in some degree to favor.

While the Greek schools taught this method of operating, Rome had no lithotomist, and Archagatus, who first proposed to cut there, was immediately banished. At length, however, the Greek operators gained footing in that city, and Celsus notices their manner of operating, but in a way so vague and obscure as to be nearly unintelligible in point of details. The precepts laid down by Celsus himself, and his method of operating, supplanted all preceding ones, and, with the exception of slight and sometimes defective modifications, was the only one practised for the extraction of calculus from the bladder, for a long lapse of centuries. During this long interval, all idea of extracting it by the natural passage had been either abandoned or forgotten.

Some time after the establishment of Christianity, medicine passed almost exclusively into the hands of the monks, and all surgical operations underwent the influence of the maxim "*Ecclesia abhorret a sanguine.*" Cystotomy fell, of course, into almost universal disuse. The barbers performed bleeding, bone setting, and little else; while the wretched sufferers from stone, condemned to perpetual anguish, continued to receive from the monks those *infallible lithontriptics* and *divine saxifrages,* which possessed no other efficacy than that of enabling them to pass through a dreary period of exhaustion, hoping for an impossible benefit to the very brink of the tomb.

At the beginning of the sixteenth century, science received a new impulse, the lay professors, otherwise styled the university doctors, became numerous, and the cutting operation was revived. The improvements it successively underwent in the hands of the surgeons of that and the following century, it is not here intended to expatiate upon. The modification of this operation, called the *apparatus major*, was practised with success in Italy by Johannes Romanus, by his pupil Marianus Sanctus,* and by Octavian de Villa, who taught it to the Collots in France ; by whom it was also practised in that country. It remained an inheritance in the family of the Collots until the beginning of the last century, when it was improved by Frère Jacques de Baulieu. His manner of operating was still further modified and improved by Cheselden, Ledran, Morand, Lecat, Louis, &c. Frère Come still further reformed this bold operation, by rendering more popular the *apparatus minor*. The zeal or genius of those great men could not, however, divest this operation of its danger, and, although it continued to be resorted to and practised with success, yet still humanity and science sighed for a milder method and more favorable results. The evil, which neither vigilance nor

* This method of operating was first described by Marianus Sanctus, and until lately has been generally ascribed to his master Joh. Romanus ; but M. Bonino, author of a Biography of the Physicians of Piedmont, has discovered, in the archives of the Faculty of Turin, documents which prove that this method was first imagined by Battista da Rapallo, who died in 1510, and who had been the master of Joh. Romanus.

temperance could avert, was far from being quietly submitted to by its victims. They murmured against a direful alternative, which imported but too frequently for them nothing more than the wretched dilemma of a never-ceasing travail on the one hand, or an expeditious martyrdom on the other. Strengthened by a natural anxiety to seek some other relief than that which the cutting operation afforded, our ignorance of the causes upon which the formation of calculus depended was sought to be removed; and accordingly air, food, drink, habits of life, atmospherical, local, or morbid influences, were subjected to the most acute and laborious inquiry.

On reviewing the works of our predecessors on this subject, while lithotomy was still ascending to its utmost height of perfection, we find here and there some ingenious but ineffectual effort to bring its brilliant though bloody reign to a close: at all events, this interesting branch of surgery did not languish in such deplorable impotency, either from apathy amongst scientific men or from despondency in the sufferers. This is abundantly evident from the eagerness with which every proposal for its improvement, however fallacious, was hailed by the public; a fact strongly attested by the liberal rewards which have not been withheld even from empiricism. Every department of science has been put to contribution, with a view to cure or relieve this dreadful complaint; materia medica, botany, chemistry, &c. have been successively appealed to for succour; and, notwithstanding the promises of philanthropists, and the dreams of alleviation to which they clung, but which they could never realize, hundreds and thousands of poor sufferers have been swept away by this unrelenting malady, without other solace in their last hours than that inspired by the credulity of enthusiasm, and a false confidence in curative means which sad experience proved to be utterly inefficacious.

The history of dissolvents furnishes a strong illustration of this point. Twice during the last century did the sufferers from stone believe themselves rid of lithotomy. Every one remembers the sweet dream into which Miss Stephens lulled them. No stone was to have resisted the omnipotence of her remedies; her praises were echoed by the first physicians in Europe; nay, the lithotomists themselves, to their honour be it spoken, swelled the ranks of her partizans. Morand wrote two Memoirs, which he presented to the Academy of Sciences in 1740, on this subject; and in the following year he published a series of cases, almost all in favour of the remedies. The confidence in their efficacy, established on the one hand by a parliamentary reward of 5000*l*, and on the other by the misguided, though well-meant, suffrages of such names as Hales, Hartley, Whitt, &c., now knew no bounds. The wonderful dissolvent, which was nothing more than prepared lime-water and soap, was quaffed with pleasure. The smallest portion of albumen, or concrete mucus, discovered in the urine, was hailed with acclamation: it was the stone that was dissolving and escaping under this inoffensive form. The enthusiasm at length became so general that the lithotomic instruments were proclaimed henceforth useless, and put under interdiction: nay, more, they were brought deridingly to trial, and a serio-comic sentence was pronounced upon them, that they should "hide their heads" for ever.

Joy, however, soon gave place to sorrow, and the sufferers learnt that they had mocked themselves with a delusion. Desperate experience reduced the eulogists to silence, and the instruments were reproduced, to the dismay of Miss Stephens and her partizans; while the deceived and unhappy patients rained on both a torrent of sarcasms and maledictions.

The second disappointment arose from the failure of the chemists. The labours of Scheele, Fourcroy, Vauquelin, Gay Lussac, Thenard, Berzelius, Prout, Marcet, and Davy, led to no other result than an accurate acquaintance with the intimate composition of calculous concretions; and the remedy of Miss Stephens, though totally inefficacious as a dissolvent of stone, did nevertheless fully establish the value of alkaline substances as a corrective of the lithic diathesis.

Another fact, no less ancient than the knowledge of the dilatability of the urethra, is the possibility of penetrating through this canal into the bladder, by means of a straight sound. The use of the straight sound was known ages ago, as is proved from their having been found in the Herculaneum; in almost all museums of antiquities; as well as upon positive documentary evidence; and, indeed, it is not improbable in the earliest times, when notions of human structure were borrowed from the dissection of brute animals, that the first attempts at passing an instrument into the bladder should have been made with a straight sound: yet this fact, so ancient, and, as we shall see presently, so frequently reproduced from time to time in the annals of science, had remained so unnoticed and unknown that, within but a few years, a distinguished surgeon of Paris, M. Amussat, proclaimed it as a discovery.*

After the Egyptians, who employed a straight pipe for the insufflation of air into the bladder, the first mention of the use of straight sounds is found in Albucasis, an Arabian writer, who gives a drawing of the instrument.†

Ambrose Paré gave directions for the division of calculi arrested in the urethra, even when placed close to the neck of the bladder. The instrument he employed for this purpose was straight. (Pl. fig. 7, 8, 9.)

In 1729, Joseph Rameau published a letter in which he extols straight sounds, and maintains, from reasons deduced from the structure of the urethra, their superiority over curved sounds.‡

The authors of the French Dictionary of Medicine and Surgery, published in 1772, maintain the opinion of Rameau.

Lieutaud speaks of straight sounds, and prefers them to the curved ones.§

At Rome, thirty years since, Professor Santarelli taught publicly that straight sounds were preferable to curved ones; and about the same time he published a Memoir at Vienna, on the method of simplifying the operation of catheterism, by the substitution of the straight for the curved sound, in which he points out the manner of effacing the first curvature of the urethra by bringing down the penis.||

Lassus, professor of the Ecole de Médecine of Paris,

* Remarques sur l'Urètre de l'Homme et de la Femme. (Extrait des Archives générales de Médecine, Dec. 1824.

† Albucasis de Chirurgia, Arab. et Lat.; edit. J. Chænning, 1778.

‡ Réflexions Anatomiques, en forme de Lettre, ou Analyse de la Dissertation de Morand, sur la Taille au Haut Appareil. Amsterdam, in 12mo. pages 6 and 7.

§ Lieutaud, speaking of the operation for puncturing the bladder, expresses himself as follows: "On peut toujours eviter cette opération, toujours dangereuse, et souvent infructueuse, parce qu'elle laisse subsister la cause de la maladie, en se servant d'une *sonde droite*, solide ou creuse. Je puis assurer sur la connaissance que j'ai de ces parties saines ou malades, qu'il n'y a aucun cas, si l'on excepte la pierre engagée dans le canal qui puisse empêcher une *sonde droite* conduite par une main un peu exercée, d'entrer dans la vessie." (Précis de Médecine pratique, tome i. p. 588.)

|| "I cateteri dritti sono conosciuti in Italia da 30 anni. Il Sig. Santarelli, prof. di ostetricia, nell arcispedale di S. Spirito di Roma, in una sua memoria intitolato 'Recerche per Facilitare il Cateterismo,' pubblicata in Vienna nel 1795, dove trovavasi di passaggio, si e ingegnato a mostrare i vantaggi che i catteteri retti hanno sugli ordinarii, facende vedere in due bellissime tavole non avere il canale dell' uretra alcuna curvatura fino alla prostata, e non stare che nelle mani del chirurgo lo far scomparire quella del disotto l'arcata del pube, abbassando il pene." (Extract from l'Osservatoire Medico of Naples, December 1825, page 187.)

taught the same doctrine but a few years afterwards; and Dr. Montaigu sustained a Thesis before the Faculty of Medicine of that city, in 1810, on the use of straight sounds.*

In 1813, Dr. Gruithuisen, of Munich, published an article in the Gazette of Saltzbourg, in which he demonstrates the facility of rectilineal catheterism.†

In 1817, and ever since, Dr. Civiale has made use of the straight sound: in 1818, the drawings of his different instruments were submitted to a commission of the Faculty of Medicine of Paris. Those instruments, as we shall see hereafter, were all straight.

In 1822, Dr. Amussat proclaimed, as a new conquest to science, the use of the straight sound. In announcing it as a discovery, (which I believe he did with the most perfect good faith,) it is evident he was ignorant of what his contemporaries, Gruithuisen and Civiale, had been engaged in long before this period.

The dilatability of the urethra, as well as the use of straight sounds, the primary elements of lithotrity, were therefore known in the earliest ages. From what follows we shall see that these data did not remain sterile in the lap of science, and that the idea itself of seizing stones in the interior of the bladder, and of extracting them through the urethra, in order to supersede the cutting operation, is by no means novel.

We would here recall to the reader's mind the bold practice of Ammon of Alexandria, who broke down the calculus in the bladder by means of a chisel introduced through the perineal incision: this method was sometimes resorted to by the lithotomists after him. Albucasis is the next who realizes this practice, but under very different and much milder circumstances.‡

* Propositions sur quelques Maladies des Voies Urinaires et sur le Catheterisme.

† "Catheterism by means of the straight sound meets with considerable opposition from the majority of medical men: nay, so much are they accustomed to the old idea that catheters must be modelled upon the direction of the urinary canal, that many of them cannot comprehend the possibility of introducing a straight sound into the bladder, and have maintained to my face that it was not possible. That which in reality exists, is possible. In two individuals upon whom I tried this method of sounding, I introduced without difficulty two glass cylinders, rounded at the extremity, and of the diameter of three or four lines, (one fourth of an inch.) I maintain, moreover, that this manner of sounding is infinitely more easy than that performed by means of curved sounds. Of this fact I have been convinced by different trials," &c. (Saltzburg, *Med. Chir. Zeitung*, 1813.)

‡ "Quod si calculus parvus sit, positusque sit in meatu fistulæ urinariæ, in eoque figatur, prohibeatque urinam ab exitu, equidem curato illum illis quæ

Fabricius Hildanus, towards the end of the sixteenth century, in speaking of the bullet forceps, which appear to have been invented by Alexander de la Croix, or by Alphonso Ferri, mentions two instruments of this kind which he had in his own cabinet,* one of which he had so modified as to render it useful for the extraction of small stones from the urethra.

Ambrose Paré had his terebra, or piercer, as we have already seen.†

Haller relates an operation of this kind performed by Fischer, in which he perforated and broke up a large-sized stone engaged in the urethra.‡

But the efforts of our predecessors to reduce calculi by mechanical means were not confined to the urethra alone. Alsaharavius, the contemporary of Albucasis, proposed, by means of an appropriate instrument, to break up small soft stones in the bladder itself.§

Sanctorius,|| who proposed, in the commencement of the præscripsimus, antequam ad sectionem perveneris; sæpissime enim ea sufficit curatio alisque sectione. Hoc etiam aliquando expertus sum, nimirum ut sumatur perforatorium ex chalybe præstanti damasceno; sit ad hanc formam, [the figure here is wanting: the drawings of the Arabs were very incorrect, and very often they are merely mentioned in the text,] triangulare sit, ad extremitatem acutum, in ligno infixum; Dein sumas filum et cum illo, ligato virgam subter calculum, ne forté in vesicam calculus revertat. Deinde intromittas ferrum perforans (terebram) cum lenitate in penis foramen donec ferrum perforans ad ipsum calculum pervenerit; et terebram cum manû tuâ revolve in ipsum calculum paulatim, paulatim, et tu conator perforationem ejus, donec illum calculum penetraveris per alterum latus. Equidem urina illico liberata erit. Diende cum manu tua, constringe reliquias calculi, ab exteriori parte virgæ illæ etenim perforatæ sunt, et cum urinâ educentur: et sanatus erit æger, si voluerit Deus excelsis." (ALBUCASIS, lib. ii. cap. lx. pag. 287. Oxonii, 1778.)

* Præter prædictam terebram Parei at tenaculam, adhuc duo alia instrumenta ad extrahendum calculum e virga, in musæo meo habeo: quorum primum ex officina chirurgicâ clarissimi viri Dn. D. Joh. Andr. a Cruce, in Latina editione, pag. 51, in Italica vero, pag. 33, depictum, et ab ipso, *Asta* appellatum, ex parte mutuatus sum; ex parte inquam, nonulla enim in ipso mutavi et correxi, uti lector, ex delineatione et descriptione videre poterit. (See Pl. fig. 10, 11, 12, 13, and 14.) Author eo quidem utitur ad extrahendos globulos plumbeos ex vulneribus sclopetorum, ego vero illud ita adoptavi, ut etiam ad extrahendos lapillos, e virga quam aptissimum sit, modo sint extra perinæum, balanum versus; qui enim in perinæo inclusi sunt lapilli, instrumentum incurvatum requirunt. (See Pl. fig. 15 and 16.)—(*De Lithotomia Vesicæ*, pag. 754.)

† Œuvres.

‡ Disputationes Chirurgicæ, tome iv. page 71 and the following.

§ Accipiatur instrumentum subtile quod nominat, *Mashaba Re bilia*, et suaviter intromittatur in virgâ et volve lapidem in medio vesicæ, et si fuerit mollis frangitur, et exibit, si vero non exiverit cum iis quæ diximus opertet incidi ut in chirurgia determinatur. (*Liber Theoricæ necnon Practicæ*, 4to. f. xciv. 1519.

|| Sanctorius Commentar. in primam fen. primi libri. Cannomensis. Avicennæ, Venet. 1626.

seventeenth century, to extract small calculi by means of a three-branched forceps, further proposed, when the stone was too large to be extracted entire, to perforate and crush it into fragments by means of a stilette.* He imagined also another instrument, which he calls a syringe, the use of which he fully explains,† and which is extremely curious, as it developes an entire method for the extraction of stone, and moreover recalls to mind the practice of the Egyptians, by the simple expression "vim vacui."

Twenty years after Sanctorius, Severinus enumerates in his book "De Efficaci Medecina," (cap. cxxxv. pars 11, de Sectionibus, page 131,) the instruments employed by different authors for the extraction of calculi. He mentions particularly the instrument of Joh. Germanus.‡

In 1561, Franco, or, more properly speaking, one of his relations, invented a four-branched instrument, which he called *Quadrupulus vesicæ:* (see Pl. fig. 21.) This instrument is the most remarkable of those we have inherited from the old surgeons. It was capable of seizing a stone as large as a hen's egg. It was, however, only destined to be introduced into the bladder through an incision in the perineum. A drawing and description of it are given in Franco's "Traité très ample des Hernies," page 147.

The mechanism of the above-mentioned instruments had even in those times, and since, been frequently reproduced under a great variety of modifications; vid. Hunter's or

* Catheterem delineat trifidum: per eum in grandiorem calculum, specillum sagittatum immittit, eo *ut putat* calculum dividit, ut fragmenta inter specilli crura cadant et possint extrahi. (See Pl. fig. 17, 18, 19.)—(Haller, *Bibliotheca Chirurgica,* tome i. page 313.)

† "Quod si calculus rupta aliqua ex papillis et per ureteres ad vesicam dejectus, spacio hebdomadæ circiter cum urinâ non rejeciatur extrahendus est ne per moram magnus evadat: quod ut fieret, excogitavimus syringam, quæ in vesicam immittenda est, quando lotio est referta: (longitudo syringæ in viro est, unius spithaminis cum dimidiâ) eâ immissâ, tunc instrumentum, quod unit tres cuspides (dum est in syringa, aliquanto plus impellitur, ut tricuspides separentur, et dilatentur: deinceps extrahitur instrumentum; quo peracto statim ab urina lapillus cum impetu ad sinum syringæ ubi est oferri solet; qui inclusus inter illas tricuspides statim extrahitur per syringam; si vero accideret quod urinæ impetus non feret lapillum ad tricuspitis sinum, tunc cum symphone per vim vacui attrahitur: in femina promptius, quia breviori syringâ eadem fieri possint." (Sanctorius, *Commentaria in primam fen. primi libri Canon.* Avicennæ, Venet. 1760, pag. 421.)

‡ Aliud mihi instrumentum extractorium proposuit Johannes Germanus, chirurgus et iatrochymicus sæpius a me, licet non satis laudatus, fistulare illud cum ternis, in extremo prehensoriis quasi digitalis interne dentatis et modice simis, recurvisque, qui dum inseritur fistula in cavum penem contracti manserit; postquam intrusus calculi locum attigit, claviculo, qui per cochleam in imo torquetur, dehiscunt, et corpusculum alienum apprehendentes rursus coarctantur, rotato cochleari scapo sic, ut revertentes organum extractum calculus sequatur.

Hale's pincers, Thomassini's, Garengeot's, Gorcy's, Earle's;* and more recently, as we shall see bye and bye, in a curved shape.

The scheme of extracting stone from the bladder through the urethra, once revived, we see gained ground, and its means of execution went on improving, until the inventors, dissatisfied with their success, gradually abandoned them, and they were ultimately eclipsed and forgotten in the consoling and specious promises of the chemists. Lithotomy was retained as the *pis aller,* or dernier resort, while the pretended efficacy of divers dissolvents began to obtain universal confidence.

It is clear, however, that, in the days of these eminent men, a knowledge of the use of straight sounds prevailed more generally than the inquiry we have in view will permit us to suppose in times more modern. And accordingly, in the next attempts at improvement which come under our consideration, we find them established upon the idea that the urethra does not admit the passage of an instrument into the bladder, unless such instrument be curved.

Daniel Episcopus has left us an instrument of this kind for the extraction of stone from the bladder.†

Mr. Elderton, of Northampton, in 1819, convinced of the possibility of crushing stone in the bladder, published, in the Edinburgh Medical Journal, a description of an instrument which he invented for that purpose. Not having by me a drawing of this instrument, I shall, as briefly as possible, give a description of it.

When this instrument was closed, it had the appearance of a thick curved sound, the external extremity of which was received into a handle. The vesical extremity was slit on its length into two branches, which were joined by a hinge; these branches were also articulated midway between the two ends of the slit, so that, when opened by a particular mechanism, the aperture represented a lozenge, into which the stone might be engaged and held fast. In the interior of the canula, a long rod of steel, carrying a flat file at its extremity, descended, and by friction was to reduce the stone to powder.

Finally, in 1821, Sir Astley Cooper published, in the eleventh volume of the Medico-Chirurgical Transactions, a case of the extraction of calculi, by means of a curved instrument which he had executed by Weiss; and subsequently, in the same Transactions, an account of several other cases in which this instrument was employed with

* Vide Medico-Chirurgical Transactions for 1821, vol. xi.

† Fabricius Hildanus, op. cit. page 755. (See Pl. fig. 15 and 16.)

success. In the first of these cases, the history of which is given by the patient himself, (the Rev. Mr. Bullen,) eighty-four calculi, some of them the size of horsebeans, were extracted from his bladder. Sir Astley speaks of his instrument, and manner of using it, in the following terms: "The instrument which I first had made for the purpose of removing these stones from Mr. Bullen were merely common forceps, made of the size of a sound, and similarly curved; but Mr. Weiss showed me a pair of bullet forceps, which he thought would, with a little alteration, better answer the purpose I had in view. He removed two of the blades of these forceps, for there were four, and gave them the form of the forceps I had constructed. The blades of this instrument could be opened while in the bladder, by means of a stylette, so as to grasp and confine the stone, and they appeared so well constructed for the purpose as to induce me to make trial of them: (see Plate, fig. 20.) On the 23d of November, 1820, I first employed them, and the manner in which they were used was as follows: Mr. Bullen was placed across his bed, with his feet resting on the floor, and a silver catheter was then introduced, and the bladder emptied of its urine. I then passed the forceps into the bladder, and was so fortunate in my first operation as to extract eight calculi." (*Op. cit.* page 359.)

These forceps I have also seen successfully employed by M. Dupuytren.

We have now seen that the idea of reducing calculi in the bladder by mechanical means was not only entertained, but endeavoured to be executed by Albucasis, Alsaharavius, Franco, Sanctorius, Paré, Fischer, the monk of Citeaux, and Colonel Martin. It will no doubt appear extraordinary, after reviewing the labours of those men for the improvement of the plan by perforation and crushing, that the two first authentic cases of success for the comminution of stone in the bladder should have been obtained by the individuals who were themselves the subjects of them, and who were both strangers to the healing art. The first, a monk of the monastery of Citeaux, was on the point of being operated upon by Hoin, an eminent surgeon of Dijon, when, terrified at the idea of the operation, he imagined a means of arriving in the bladder with a statuary's chisel, by introducing it through a flexible hollow sound. The chisel was a straight steel cylinder, sharpened at the extremity destined to act upon the stone. When this instrument was introduced, and brought in contact with the stone, the monk, by striking gently on the outer end of the chisel, detached small fragments, which were carried out by the stream of urine. By this means, in the course of a year, he reduced the calculus entirely, and took great pleasure in showing to the curious the fragments carefully preserved in a small box. The second case, that of Colonel Martin, was one of only partial success, as will be evident on perusing the following extract from the Edinburgh Medical and Surgical Journal, for January 1828, p. 221.

" As the exact date and the precise circumstances of the case of Colonel Martin appear to be viewed with doubt by the authors of the Report on the work of M. Civiale, we give the following account, communicated verbatim by Dr. Monro from his father's manuscript lectures, in which we have seen the case. This reference proves indisputably that the date of Colonel Martin's operation was not later, whatever sooner, than 1800.

" Before I conclude this subject, says Dr. Monro, it may be worth while to mention the case of an officer of high rank in India (Colonel Martin), who persuaded himself that he might be able to rid himself of a stone in his bladder by rasping it and cutting it with a file. He accordingly procured a steel sound, of the ordinary length and shape, and about one tenth part of an inch in its diameter, which was made rough like a file on its convex part and sides for the length of three quarters of an inch from its point. He, with much courage and perseverance, introduced this into the bladder four or five times a day, filing the stone for half an hour or so each time, and persevered in doing so for nine months. During that time he rasped off a great deal of powder, and cut off a number of small pieces from the stone, several of which he sent me in a letter under cover to the Right Hon. Sir J. Sinclair, along with the instrument he had employed. He was unfortunately seized, at the time he wrote me, with a liver complaint, of which he died soon after, without having destroyed or discharged the whole stone.

" It appears that the stone had been in general lodged within and grasped by the sphincter of the bladder, in which situation he could reach it, and generally file it, without its slipping backwards into the cavity of the bladder.

" Notwithstanding the progress the colonel had made in his case, I apprehend there are few readers who would think his method should be prosecuted, and still fewer who would have the courage to practise it."

I will add one reflection upon Colonel Martin's case: it is evident that his stone was not in the interior of the bladder, but either in the prostatic, or farther forward in the membranous portion of the urethra, and consequently it must be classed with the cases of Ambrose Paré, Fischer, &c. and not with that of the monk of Citeaux.

I have now brought down my subject to our own times, and detailed all the circumstances that bear upon it. We have seen, in the progress of this inquiry, that the dilatability of the urethra, as well as the use of straight sounds, were known in the remotest times. We have seen, from the labours of the surgeons of the middle ages, that the crushing and extraction of stone was not deemed by them an impossibility. We have seen more than one amongst them propose a straight tube, containing another tube with branches to expand in the bladder, there to seize a stone, and a stilette to crush or divide it. Here is the theory of lithotrity, quite perfect; and, nevertheless, lithotrity is a curative method entirely new: for, however ingenious the old surgeons were, their instruments never went beyond the sphere of mere project. Sanctorius makes no mention of the application of his instrument. Severinus does not say that Germanus used his instrument. Neither can it be said that Daniel Episcopus, Hales, Sir A. Cooper, or Mr. Elderton, have contributed to the invention of lithotrity; their sounds being curved, and only adapted to the extraction of small stones. Still less did the means employed by the monk of Citeaux and Colonel Martin lead to this discovery, as they are too dangerous and too difficult of application to become models of imitation. Lithotrity is, therefore, an entirely new curative method for stone. To be convinced of this it is only necessary to compare its results with those obtained by the means in use before it was made known to the world. We have seen that its elements lay, some of them thinly scattered and almost forgotten in the domain of science, no person, until our own times, having collected them into a method at once complete and applicable.

We have on purpose refrained from classing the labours of Gruithuisen with those of his predecessors, on account of the efforts made by a French surgeon, who has lately turned his attention to this subject, to fasten upon him the merit of this invention. We shall examine with strict impartiality, and with a full sense of the esteem which this learned man deserves, his claim to this honour. We have no reputation to demolish or to build up by the depreciation of the services which Dr. Gruithuisen may have rendered to science, and we enter upon this discussion resolved to do justice.

What had been done for lithotrity before the time of Dr. Gruithuisen, we have already seen. We shall proceed to consider in what manner this distinguished individual advanced the state of science.

It was not by the employment of straight sounds: they were known from time immemorial.

It was not by proving the possibility of introducing thick sounds into the urethra: this fact has been known ever since strictures of the urethra have been treated by surgeons.

It was not by the originality of the idea of crushing stone in the bladder by mechanical means: the names, already so often mentioned, of Albucasis, Alsaharavius, &c. lay equal claim to this honour.

In fine, was it by better means of execution than those proposed by his predecessors that Dr. Gruithuisen's claim to the invention of lithotrity will be sustained? A single glance at the drawings which he has published in the Gazette of Saltzburg will suffice to convince any man, at all conversant with the operation, of the utter impracticability of crushing a stone in the bladder with such an instrument. In order to perform this operation, the stone must, in the first place, be laid hold of, and fixed so firmly that the surgeon may have nothing to fear from its slipping during the drilling process. Let us now see if Dr. Gruithuisen has fulfilled this indispensable condition of lithotrity.

About a century ago Marini proposed a wire noose, or piano cord, for the purpose of extracting small calculi from the urethra. This noose was to be passed behind the stone, much in the same way as we proceed when we attempt the extraction of a cork from the lower part of the neck of a bottle. This method has been employed about a year ago, with success, by Dr. Rousseau, of Paris. The wire noose which Marini employed for urethral calculi, Dr. Gruithuisen thought possible to apply to those in the interior of the bladder:* but he had not reflected that the mobility of the caculi would have formed an insurmountable obstacle to the employment of the means he proposes: besides, in the bladder, he could not place the stone in the noose with his finger, as he might have done in the urethra. Admitting even that the noose may seize the stone in the bladder, it is still obvious that it cannot be held with sufficient firmness at the end of an open canula, to support the action of a

* See Pl. fig. 26. All his instruments represented in the plate have been copied from the plate in the German journal.

drill. Besides, the importance of preserving the bladder from violence or injury must not be overlooked. Now, in what manner will this wire noose protect the organ against the action of his trephine or his lance-pointed stilette? Neither Dr. Gruithuisen nor any other man in his senses would have trusted to it.

Such is Dr. Gruithuisen's instrument for seizing and fixing the calculus. His next instrument is a crook, (see Plate, fig. 29,) with which he proposes to crush the fragments. To employ this is obviously impossible. By what means are those fragments to be fixed, before this crook can be brought to act upon them? Dr. Gruithuisen himself, notwithstanding the truth of his theory, must have been convinced of the inutility, for any practical purpose, of the instruments he proposed, as he never so much as attempted to obtain any of the results of which he supposes the facility so great. To place this point in a still stronger light, let Gruithuisen's instruments be compared with the instruments proposed by the older surgeons, and it will be obvious that the bullet pincers of De la Croix and Ferri, the forceps of Fabricius Hildanus, Germanus, Sanctorius, &c. were better calculated to point out the way for the invention of proper means for seizing and fixing the stone than Marini's wire noose, which Gruithuisen has reproduced; while the monk of Citeaux's chisel, and the grinder of Paré and Franco, are, to say the least of them, as advantageous as Gruithuisen's trephine, or lance, which, after all, had been proposed by Albucasis. It is now time to ask, what are the great improvements of the ancient instruments upon which Dr. Gruithuisen's claim as the inventor of lithotrity may be supported? He has not proposed any thing more applicable than his predecessors had done; nay, his instruments in some respects are not so perfect as theirs. He has neglected to reproduce in his Memoir all the facts with which those men had enriched science, although he admits he was not unacquainted with their works.* He affirms that, with a straight sound, he can determine geometrically the size of the stone; that a dilatation of the urethra capable of admitting a sound from six to eight lines in diameter will suffice for the employment of his instruments. These assertions are certainly calculated to startle us.

He further proposes, for the destruction of calculus in

* The heading of his Memoir is, "Are we to abandon the hope entertained in former times of being able, at some period or other, of destroying stone in the bladder, either by mechanical or chemical means?"

the bladder, the perfusion of a quantity of some solvent liquid, introduced into the bladder by means of the double sound of Hales, modified by himself. The fluid he directs from the second story of the house of the patient. And, finally, he proposes galvanism, and the dissolving properties of this agent should have dispensed with all further research on this subject, as he assures us the hardest stones *will melt like butter* when subjected to a battery of from 600 to 1000 couples.

We shall bring the consideration of Dr. Gruithuisen's claims to a close by a quotation from the Reporters of the Academy of Sciences, pages 20, 21: "But, above all, it was necessary to mature and arrange those ideas which Dr. Civiale believed to be entirely his own, while views, if not identical, at least analogous, had been published in 1813, in a German Gazette, of the existence of which he must have been altogether ignorant. There, to his great astonishment, he discovered that the *initiative* of his lithontriptic system belonged to Dr. Gruithuisen, who had preceded him; and it was only necessary for him to cast his eyes over the plates and descriptions of those instruments, though rude and merely imaginary, to show him that he must take his rank after him, notwithstanding that he had himself discovered all, and without borrowing from any one else. But, if M. Civiale is modestly satisfied with the second place with respect to the vague, incoherent, though ingenious, plan of the Bavarian doctor, we are of opinion that he deserves the first place for the happy, and we may say learned, manner by which he has established, developed, and reduced to practice, a project which had been merely hinted at in a foreign journal; which remained uncultivated and forgotten in the country which gave it birth; altogether in mere theory and speculation, having never had the least attempt at execution, either in its instruments or in its application to practice."

It may be safely concluded, therefore, that Dr. Gruithuisen is not the author of lithotrity. It would, however, be unfair not to admit that he was aware of the possibility of crushing stone in the bladder. Indeed, I am not aware that Dr. Gruithuisen, whose claims are clear as far as they go, has ever set up any pretension to be considered the inventor. Officious and interested persons have placed him in this position, with a view to diminish the merit of the real inventor, Dr. Civiale. If in this discussion we have dissented from a claim which Gruithuisen has never himself asserted, it by no means takes away from the respect we entertain for him as a philanthropist and a truly enlightened member of the medical profession.

All those attempts, ancient as well as modern, ingenious no doubt as they are, were however but the prelude to the invention of lithotrity. To whom, then, are humanity and science indebted for this long-sought for and important benefit? The instrument, in its present simple and perfect state, the operative process, as well as its successful application, are all due to the same distinguished individual, Dr. Civiale.

[To be continued.]

---

